# A method for screening salt stress tolerance in Indian mustard (*Brassica juncea*) (L.) Czern & Coss at seedling stage

**DOI:** 10.1038/s41598-024-63693-6

**Published:** 2024-06-03

**Authors:** Garima Aggarwal, Premnath Edhigalla, Puneet Walia, Suruchi Jindal, Sanjeet Singh Sandal

**Affiliations:** 1 Department of Genetics and Plant Breeding, School of Agriculture, Lovely Professional University, Phagwara, Punjab India; 2 Department of Molecular Biology and Genetic Engineering, School of Bioengineering & Biosciences, Lovely Professional University, Phagwara, Punjab India

**Keywords:** Plant sciences, Plant breeding, Plant stress responses

## Abstract

Fifty-nine diverse *Brassica juncea* (Indian mustard) genotypes were used to find an effective screening method to identify salt tolerance at the germination and seedling stages. Salinity stress limits crop productivity and is difficult to simulate on farms, hindering parental selection for hybridization programmes and the development of tolerant cultivars. To estimate an optimum salt concentration for screening, seeds of 15 genotypes were selected randomly and grown in vitro at 0 mM/L, 75 mM/L, 150 mM/L, 225 mM/L, and 300 mM/L concentrations of NaCl in 2 replications in a complete randomized design. Various morphological parameters, viz., length of seedling, root and shoot length, fresh weight, and dry weight, were observed to determine a single concentration using the Salt Injury Index. Then, this optimum concentration (225 mM/L) was used to assess the salt tolerance of all the 59 genotypes in 4 replications while observing the same morphological parameters. With the help of Mean Membership Function Value evaluation criteria, the genotypes were categorized into 5 grades: 4 highly salt-tolerant (HST), 6 salt-tolerant (ST), 19 moderately salt-tolerant (MST), 21 salt-sensitive (SS), and 9 highly salt-sensitive (HSS). Seedling fresh weight (SFW) at 225 mM/L was found to be an ideal trait, which demonstrates the extent to which *B. juncea* genotypes respond to saline conditions. This is the first report that establishes a highly efficient and reliable method for evaluating the salinity tolerance of Indian mustard at the seedling stage and will facilitate breeders in the development of salt-tolerant cultivars.

## Introduction

Several abiotic stresses, such as extreme temperatures, drought, floods and soil salinity, have negatively impacted the growth and cultivation of agricultural crops. Among these, soil salinity is particularly destructive, leading to significant decreases in arable land, crop yield, and quality^1–3^. In coastal areas, salinity is caused by ocean water influx, whereas inland salinity is caused by inadequate irrigation practices and high salt deposition in ground water^4–6^.Salinity affects around 33% of irrigated agricultural fields and 20% of all cultivated agricultural fields globally and such regions are increasing at an alarming rate of 10% every year due to a variety of factors such as weathering of rocks, low precipitation, irrigation with saline water, excessive surface evaporation, and inefficient cultural practices^7,8^. Soil salinity impacts crop physiology along with various molecular and biochemical functions. It affects seed germination and seedling growth while inhibiting root, shoot elongation and dry matter accumulation^9–11^. Osmotic stress is the first stress a plant encounters when exposed to saline conditions which immediately slows down the rate of germination and subsequent plant growth^12–14^. Excessive salinity has negative effects on plant photosynthetic activities, causing leaf injury, chlorosis, and senescence^15^. Due to the high concentration of soluble salts in the soil, such as Ca^2+^, Mg^2+^, Na^+^, and Cl^–^, soil water availability decreases causing nutritional imbalance and production of toxic ions affecting the plant growth^16,17^. Moreover, plants affected by salt stress are more susceptible to disease outbreaks^2^. Several comprehensive analyses on techniques for addressing the salinity issue highlighted two main approaches: the reclamation of salt-affected soils through the application of chemical additives, or the use of saline soils for cultivating salt-tolerant plants^18–21^. The latter approach is more practical considering demonstrated availability of adapted genetic variation in various species.

Brassicas are the third-most important edible oil crop in the world, ranked after soybean and palm oil^22^. Higher salt tolerance is exhibited by amphidiploid Brassica species like *B. carinata, B. juncea*, and *B. napus* compared to their diploid progenitors, *B. oleracea, B. nigra, and B. rapa*^23^. Among them, *B. juncea* also known as Indian mustard was grown extensively in India and China until the late 1950s, when *B. napus* became more productive. New to Australia (1980s) and Canada (2002), it has substantial morphological and physiological heterogeneity^24^ and stands out as one of the most essential oil-yielding crops in salinity affected soils^25^. Given that the salt tolerance level in *B. juncea* remains consistent throughout its entire life cycle, selecting for salt tolerance during the earliest development stages might yield individuals which are tolerant at all subsequent growth phases^19,26^. Earlier studies have used various morphological traits of seedlings as well as biochemical and physiological traits ranging from levels of osmoprotectants, K^+^/Na^+^ ratio, total osmolytes, photosynthetic rate, water use efficiency rate and total chlorophyll content to assess salinity tolerance^27–32^. In recent times, morpho-physiological traits have been observed at the germination stage not only with the aim of screening salt tolerance but also to determine a reliable trait or indicator in different species. These studies have utilized a mathematical approach to screen for salinity stress known as Fuzzy set theory, it provides a framework for capturing the gradual and multifaceted nature of plant response to stress enabling a more nuanced understanding of complex traits as salinity and drought tolerance^33^. Root shoot length and shoot fresh weight have been reported as indicators for salt tolerance in *B. napus*^34,35^, germination index along with germination vigour index in sunflower^36^, seedling vigour index in rice^37^ and root length in chickpea^38^. Multiple studies exist that explore salinity tolerance in Indian mustard; however, there is a noticeable gap in the literature concerning the identification of a single reliable trait for assessing salinity tolerance in *Brassica juncea* during the germination stage^39–43^. It is crucial to identify an effective and reliable trait for the assessment and screening of salt-tolerant lines for breeding salt tolerant varieties. It would be one step forward for breeders to screen large number of germplasm at the seedling stage itself and thus in this study, we investigate the salt tolerance of 59 *B. juncea* genotypes at different salinity levels to establish an effective salt concentration at which the germplasm can be screened for salt stress tolerance and a reliable screening parameter.

## Methods

### Experimental material

There were two main objectives of this study, first to determine the optimum salt concentration for screening salt tolerance and second to determine a reliable screening trait for salinity tolerance at the germination/seedling stage. For this purpose, seeds of 59 *Brassica juncea* genotypes were obtained from Lovely Professional University, these are a set of diverse released genotypes adapted to Indian sub-continent conditions. The experiment was conducted in 2023 using a completely randomized design (CRD) at the Lovely Professional University Plant Breeding Laboratory, wherein seeds were grown in vitro in petri plates in a growth chamber while maintaining a temperature of 28 °C during the day and 23 °C at night with humidity ranging from 50 to 70%.

### Determination of an optimum NaCl concentration

Initially, to establish the ideal salt concentration, a total of fifteen genotypes were chosen at random on which four different salt concentrations were used^35,36^. The control treatment in this experiment consisted of distilled water (0 mM/L) and a range of four different NaCl concentrations (75, 150, 225, and 300 mM/L) were utilised. A selection of twenty seeds was made from each genotype, ensuring that they displayed uniformity and good health. These seeds were subjected to surface sterilisation using 15 mL of 70% ethanol for a duration of 15 min. Following this, they were rinsed thoroughly using distilled water. The seeds were then immersed in pure water for a period of 12 h. These seeds were then evenly distributed in petri plates using two layers of blotting paper as base. Seeds from two genotypes with the same NaCl concentrations were placed on either side of each petridish, which was divided into two halves using a thread. Each petri dish was then filled with 12 mL of either distilled water (control) or one of the aforementioned NaCl solutions. To prevent evaporation, the petri dishes were covered with a lid and kept in growth chamber with a constant 12 h of light and dark periods, at a temperature of 28 °C during the day and 23 °C at night. Two biological replicates were used in this stage of the experiment. Each petri dish was examined every two days, and using a dropper, the old solution was replaced with an equal amount of the new solution. In the control treatment, the same procedure was used to replace the old distilled water with new. When the length of a seed's radicle reached or went over 2 mm, it was considered that the seed had germinated. Various morphological parameters like rate of germination, root length, shoot length, seedling length, seedling fresh weight and dry weight were measured as mentioned below. The appropriate salt concentration was determined by assessing the salt-injury index (SII) for each parameter, which was regarded to be 0.5 to that of control. In other words the salt concentration was considered to be the best-fit concentration when the salt injury index (SII) was 50% of the control because at this point the seedlings begin to show the extent of damage or injury caused by salt stress on a plant^35^.

Rate of germination (G) was calculated using following formula^36^;

$$\text{G}= \frac{{\text{G}}_{\text{t}}}{\text{T}} \times 100{\%}$$

where ${\text{G}}_{\text{t}}=\text{Number \, of \, seeds \, germinated }7\text{ DAS},\text{ T}=\text{Total \, seeds \, sown},$(DAS = days after sowing/placing).

Measurements for Lengths of root (RL), shoot (SHL) and seedling (SL) were taken in centimetres and for Fresh weight of seedling (SFW) in milligrams after 7 DAS.

Measurements for Dry weight of seedling (SDW) were taken individually in milligrams following uniform drying at 70 °C until a constant weight was attained.

Salt Injury Index (SII) and Salt Tolerance Index (STI) were calculated as follows:

$$\text{STI }=\frac{\text{Value \, of \, trait \, under \, stress}}{\text{Value \, of \, trait \,  under \, control \,  condition}}$$

STI was calculated for each parameter to eliminate the effect of genotypic background^34,35^.

$$\text{SII }=1-\text{value \, of \, STI \, of \, each \, trait}$$

### Determination of tolerance at optimum salt concentration

**Figure 1:**
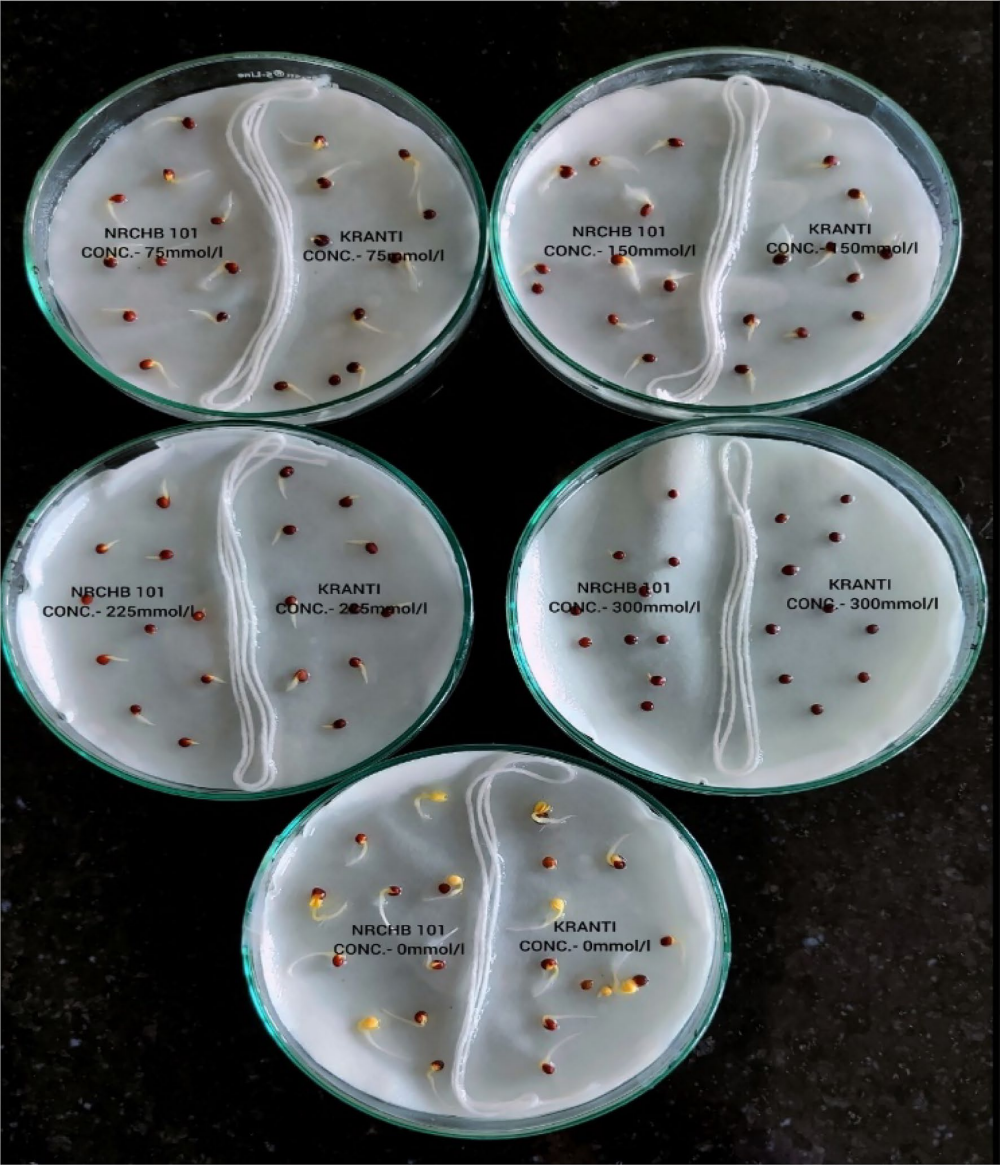
Germination of the genotypes #7 and #10 at various levels of salt stress concentration.

The salt tolerance of all 59 *B. juncea* genotypes was determined using the optimum salt concentration (225 mM/L) determined in first experiment. Ten healthy seeds of the same genotype were placed in each half of each 9 cm petri dish, which was divided into two equal portions and treated with the 225 mM/L NaCl solution (Fig. 1). A separate control treatment with a concentration of 0 mM/L was also maintained. The biological replicates of each genotype were four. Each of the four replications, as well as the control, contained ten seeds each. A similar sterilisation procedure was carried out before administering NaCl solutions, as previously mentioned. The petri dishes were monitored on a regular basis, and old solution was replaced with new solution. To evaluate salt tolerance of all *B. juncea* genotypes, same morphological parameters as mentioned above were determined.

The estimation of salt tolerance levels was conducted by utilising the fuzzy comprehensive evaluation approach, which relied on the membership function value^44^. The MFV was determined as follows:

$${\text{X}}_{\text{i}}= \frac{\left(\text{X}-{\text{X}}_{\text{min}}\right)}{\left({\text{X}}_{\text{max}}- {\text{X}}_{\text{min}}\right)} \times 100\%$$

where $${\text{X}}_{\text{i }}=\text{MFV \,  of \, a \, trait},\text{ X}=\text{STI \, of \, a \, trait}, {\text{X}}_{\text{max}}=\text{Maximum \,  STI \, value}, {\text{X}}_{\text{min}}=\text{Minimum \,  STI \, value}$$

Mean MFV values were calculated for each trait for all the genotypes. Range of mean MFV was from 0 to 1 and the higher MFV depicts higher salt tolerance.

### Hierarchical cluster analysis

Furthermore, using hierarchical cluster analysis the genotypes were categorised into the following groups as per their salt tolerance levels:

**Table:** 

Group	Salt tolerance grades/levels
1	Highly salt tolerant (HST)
2	Salt tolerant (ST)
3	Moderately salt tolerant (MST)
4	Salt sensitive (SS)
5	Highly salt sensitive (HSS)

The mathematical evaluation model of salt tolerance was developed using SPSS software. This model is represented by the following equation

$$\text{Y}=\upmu +{\upbeta }_{\text{RL}}\, {\text{X}}_{\text{RL}}+{\upbeta }_{\text{SHL}}\, {\text{X}}_{\text{SHL}}+{\upbeta }_{\text{SL}}\, {\text{X}}_{\text{SL}}+{\upbeta }_{\text{SFW}}\, {\text{X}}_{\text{SFW}}+{\upbeta }_{\text{SDW}}\, {\text{X}}_{\text{SDW}}$$

where $$\upmu =\text{ constant \, denoting \, the \, random \, error \, term},\text{ Y}=\text{mean \, MFV \, of \, genotype}, {\beta }_{i}=\text{unstandardized \,  coefficient}$$

### Statistical analysis

ANOVA was performed to test the level of significance amongst different concentrations. Correlation analysis between different traits was performed using IBM SPSS V.25. Using the mean MFV and STI of different traits a linear regression analysis was performed along with a mathematical evaluation model was developed by performing multiple regression analysis using SPSS software (SPSS, Chicago IL, United States).

### Research involving plants

Experimental materials used in this study are cultivated varieties available in public domain and the experiment complies with relevant institutional, national, and international guidelines and legislation. No field trials were conducted.

## Results

### Establishment of appropriate concentration of salt stress

**Figure 2:**
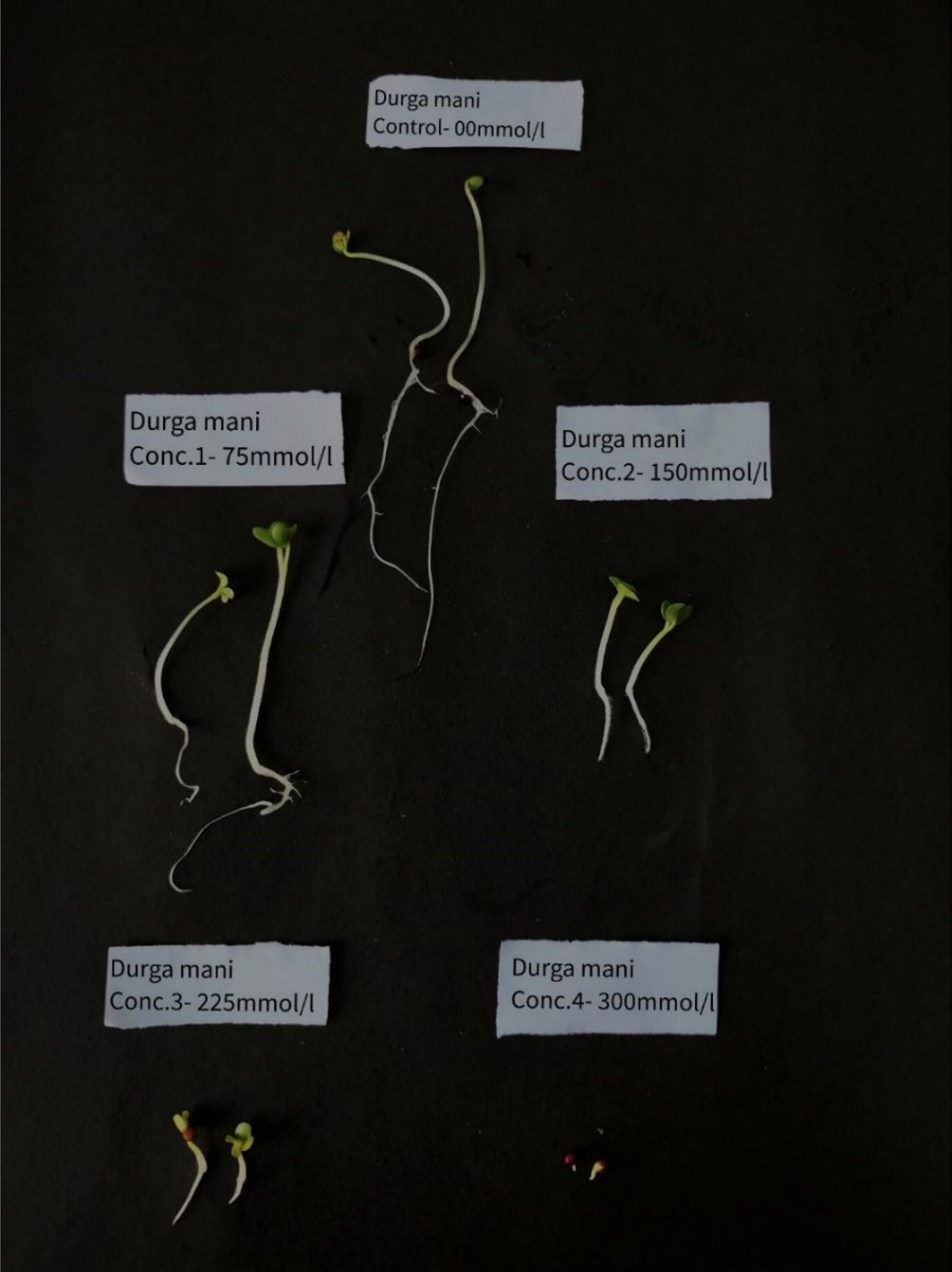
The germination of #15 genotype under different salt stress concentration on the seventh day.

To determine the optimum salt concentration for screening the salt tolerance of *B. juncea* genotypes, 15 genotypes were randomly selected from the experimental material. After 7 days, data pertaining to the RL, SHL, SL, SFW, SDW, and GR of 15 *B. juncea* genotypes was collected, demonstrating the negative effect of salt stress on developing seedlings (Fig. 2).

The optimum concentration of salt stress can be assessed using the salt injury index (SII). SII provides an estimate of how much damage salinity stress causes to plants. It is the threshold at which half of the plants begin to show negative effects from salt stress. In this experiment, the salt concentration at which SII is 0.5 is regarded as the ideal concentration as the half of the seedlings show damage caused by salt stress, allowing the salinity tolerance to be characterised. Higher the SII, greater will be the damage caused by salinity stress.

**Figure 3:**
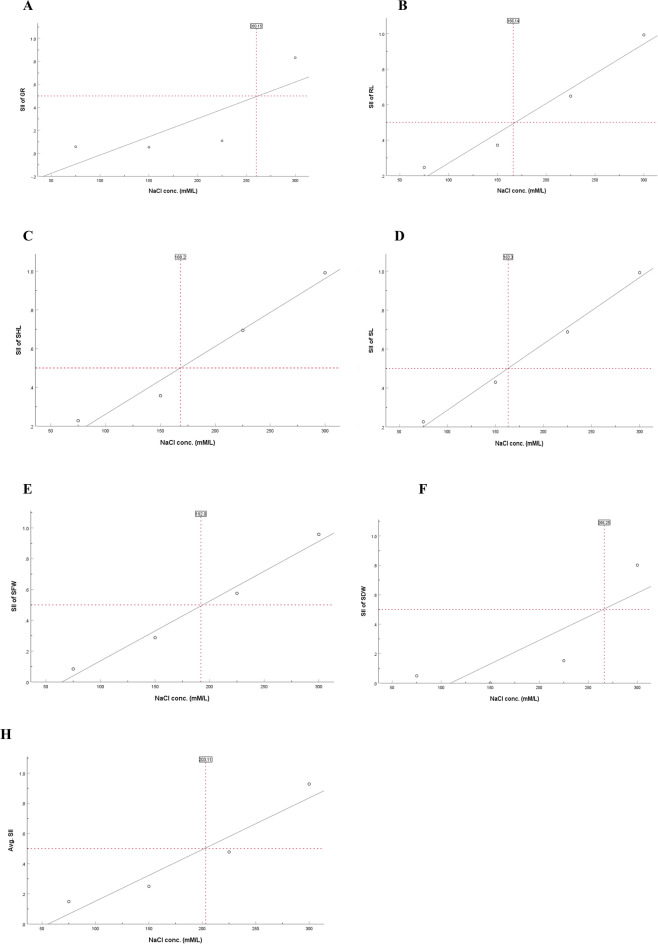
Selection of optimum salt concentration for evaluating salt tolerance ability of 59 *Brassica juncea* genotypes when salt injury index (SII) was reduced to 50% of the control. Linear fit between SII of each parameter against different NaCl concentrations (**A**) SII of germination rate vs NaCl (mM/L) (**B**) SII of Root length (RL) vs NaCl (mM/L) (**C**) SII of Shoot length (SHL) vs NaCl (mM/L) (**D**) SII of Seedling length (SL) vs NaCl (mM/L) (**E**) SII of Seedling fresh weight (SFW) vs NaCl (mM/L) (**F**) SII of Seedling dry weight (SDW) vs NaCl (mM/L) (**F**) Average SII vs NaCl (mM/L).

Based on the recorded data, SII of each trait was calculated (Table S1). The SII of the morphological traits across all 15 *B. juncea* genotypes for each trait was subjected to a linear regression analysis (Fig. 3). For GR, 0.5 measure of SII was recorded at 260 mM/L NaCl treatment. Similarly, for RL, SHL, SL, SFW, and SDW, the SII of 0.5 was recorded at salt concentrations of 166.14, 168.2, 163.38, 192, and 266 mM/L respectively. The 0.5 average SII of all the morphological traits under study was recorded at 203 mM/L. Therefore, 225 mM/L NaCl treatment was considered as optimum salt stress concentration.

### Analysis of correlation among physiological traits under stress

Using the determined optimum salt concentration (225 mM/L), the morphological data of the following traits namely, GR, RL, SHL, SL, SFW, and SDW of each genotype was measured (Table S2). STI value can be used for evaluating the impact of NaCl on salt tolerance parameters of *B. juncea* genotypes as it measures the ability of a plant to withstand and thrive in saline conditions. It is determined by contrasting the performance of plants in salinized and non-salinized (control) environments. Higher STI value indicates lesser impacts while lower value indicates more impact. STI values determined for the morphological traits under study are shown in the supplementary material (Table S3). A correlation analysis was performed to show the association between the morphological traits (Table 1). STI of all the morphological traits recorded were found to be positively correlated with each other. A high positive correlation was found between STI of RL and SHL (0.940), and STI of SHL and SL (0.935) which indicates the ability of the seedlings to tolerate salt stress by improving their morphological traits. Correlation for STI of SHL and SFW was 0.820, while the correlation between the STI of SL and SFW was 0.816. The weakest correlation coefficient (0.385) was found between STI of RL and SDW.

**Table 1:** Correlation coefficient analysis of Salt tolerance index for each parameter of 59 *Brassica juncea* genotypes at germination stage under optimum salt stress condition.

	*STI*_*RL*_	*STI*_*SHL*_	*STI*_*SL*_	*STI*_*SFW*_	*STI*_*SDW*_	*STI*_*GR*_
STI_RL_	1					
STI_SHL_	0.768**	1				
STI_SL_	0.940**	0.935**	1			
STI_SFW_	0.722**	0.820**	0.816**	1		
STI_SDW_	0.385**	0.549**	0.489**	0.669**	1	
STI_GR_	0.494**	0.696**	0.627**	0.753**	0.707**	1

### Hierarchical cluster analysis

**Figure 4:**
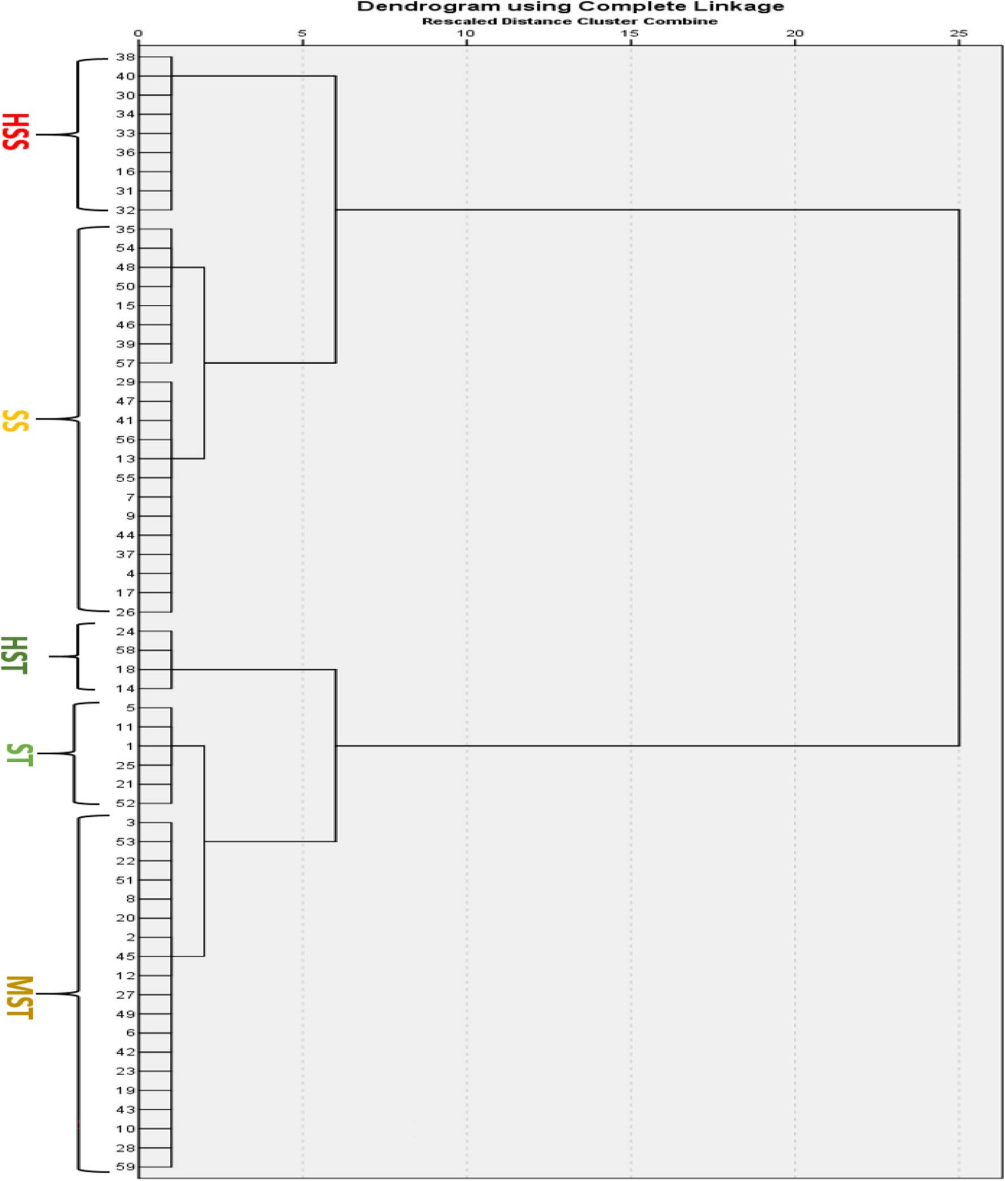
Hierarchical clustering of 59 *Brassica juncea* genotypes based on their mean membership function values (Mean MFV) where *HSS* Highly salt stressed, *SS* Salt stressed, *MST* Moderately salt tolerant, *ST* Salt tolerant, *HST* Highly salt tolerant.

**Figure 5:**
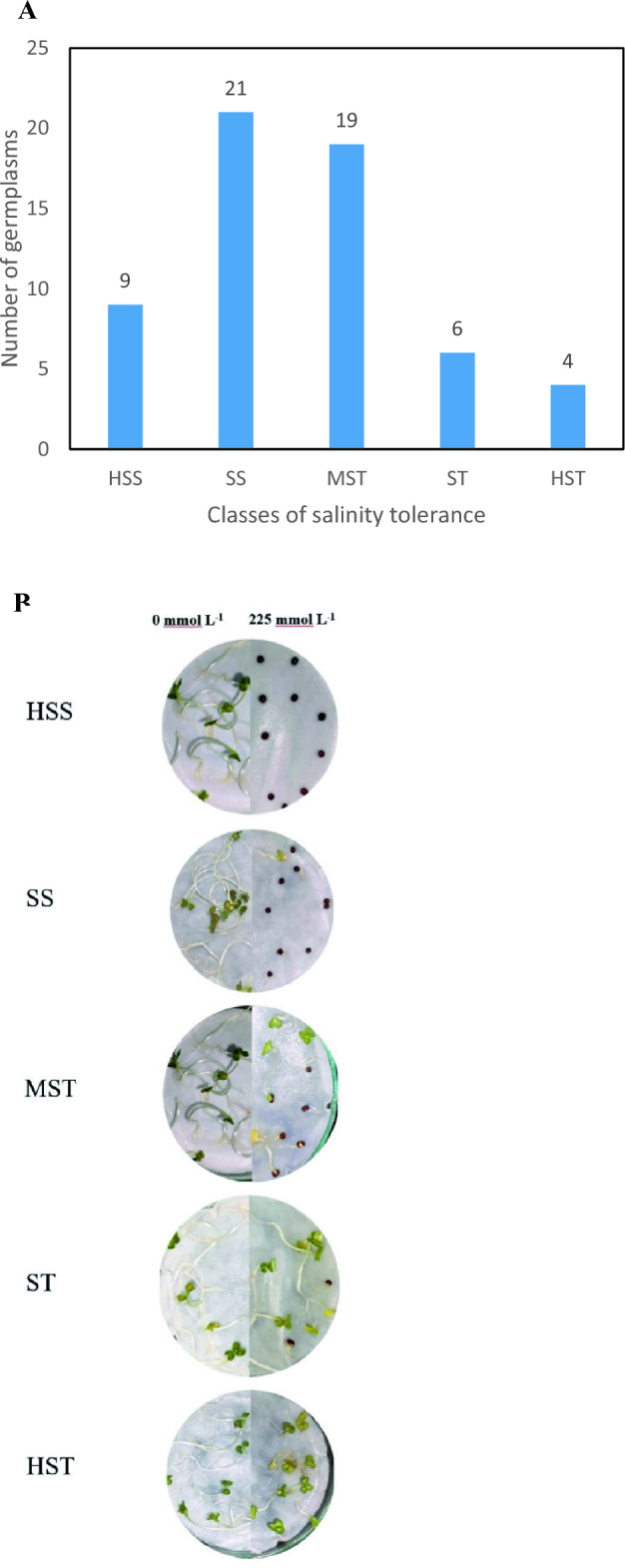
Classification of 59 *Brassica juncea* genotypes on the basis of mean membership function value (mean MFV). (**A**) Categorization of 59 *Brassica juncea* genotypes according to salt tolerance based on mean MFV. (**B**) Comparison between control (0 mM/L) and stress condition (225 mM/L) at various salt tolerance categories where *HSS* Highly salt stressed, *SS* Salt stressed, *MST* Moderately salt tolerant, *ST* Salt tolerant, *HST* Highly salt tolerant.

Fuzzy comprehensive evaluation method was used to obtain the MFV and mean MFV of each indicator. A higher mean MFV indicates stronger salt tolerance. A hierarchical cluster analysis based on the Furthest Neighbor was utilized to classify 59 genotypes into five categories based on their mean MFV values (Fig. 4). Out of 59 *B. juncea* genotypes, 4 genotypes were classified as HST, 6 genotypes as ST, 19 genotypes as MST, 21 genotypes as SS, and 9 genotypes as HSS (Fig. 5).

### Establishing a mathematical model for salt tolerance evaluation

The mean MFV and STI of each morphological trait was analyzed using multiple regression analysis to establish a mathematical model for evaluating the salt tolerance. The regression equation derived is given as: Y = 0.027 + 0.207 × STI_RL_ + 0.362 × STI_SHL_ + 0.328 × STI_SL_ + 0.345 × STI_SFW_ + 0.130 × STI_SDW_ (P < 0.01) (Table 2). In this equation values of unstandardized coefficients of STI of RL, SHL, SL, SFW and SDW are 0.207, 0.362, 0.328, 0.345 and 0.130 respectively. µ (random error term) was 0.027. Y indicates the salt tolerant ability of a certain *B. juncea* genotype.

**Table 2:** Multiple regression analysis for salt tolerance indices of root length (STI of RL), shoot length (STI of SHL), seedling length (STI of SL), seedling fresh weight (STI of SFW), and seedling dry weight (STI of SDW) in the presence of 225 mM/L NaCl concentration.

Coefficients^a^								
Model		Unstandardized Coefficients		Standardized Coefficients	t	Sig	99.0% confidence Interval for B	
		β	Std. Error	β			Lower bound	Upper bound
1	(Constant)	0.027	0.008		3.593	0.001	0.007	0.047
	STI RL	0.207	0.187	0.132	1.107	0.273	-0.292	0.706
	STI SHL	0.362	0.161	0.263	2.250	0.029	-0.068	0.792
	STI SL	0.328	0.332	0.214	0.989	0.327	-0.558	1.214
	STI SFW	0.345	0.042	0.287	8.294	0.000	0.234	0.456
	STI SDW	0.130	0.013	0.230	9.973	0.000	0.095	0.164

^a^Dependent Variable: Mean MFV.

*Y* = 0.027 + 0.207 × STI_*RL*_ + 0.362 × STI_*SHL*_ + 0.328 × STI_*SL*_ + 0.345 × STI_*SFW*_ + 0.130 × STI_*SDW*_*.*

### Verification of the mathematical evaluation model of salt tolerance

To verify the accuracy and usefulness for predicting the salt tolerance using the established mathematical model, three genotypes were randomly selected from each salt tolerance category to calculate the Y values (Table 3). The Y values were derived for each genotype by substituting the morphological data in the mathematical model and the resultant Y values were compared with their respective mean MFV of each genotype. For example, the Y value of the #48 (SS) genotype is 0.22, and its mean MFV is also 0.22; the Y value of the #18 genotype is 0.70, and its mean MFV is also 0.71, indicating that the mean MFV and Y values are very close. Higher mean MFV value indicates high salt tolerance. The obtained results have demonstrated that the formula can be used to determine any *B. juncea* genotype salt tolerance at the germination stage. The *B. juncea* genotypes chosen to determine the ideal salt concentration can also be estimated to have salt tolerance evaluation using this model (Table S5). Therefore, STI based Y value estimation of seedling growth parameters is accurate for predicting the salt tolerance of *B. juncea* genotypes (Table S6).

**Table 3:** Multiple regression analysis with Mean Membership function value (mean MFV) for salt tolerance verification.

Genotype no	STI of RL	STI of SHL	STI of SL	STI of SFW	STI of SDW	Mean MFV	Y value
33	0.018	0.021	0.020	0.049	0.115	0.062	0.076
36	0.025	0.031	0.029	0.041	0.178	0.068	0.090
16	0.024	0.038	0.029	0.064	0.228	0.082	0.107
48	0.074	0.118	0.101	0.104	0.575	0.229	0.229
55	0.103	0.161	0.136	0.222	1.027	0.359	0.361
13	0.182	0.294	0.227	0.195	0.436	0.361	0.370
43	0.078	0.354	0.180	0.332	1.020	0.463	0.478
59	0.238	0.266	0.252	0.304	0.846	0.480	0.470
20	0.156	0.322	0.230	0.459	0.998	0.536	0.539
1	0.288	0.440	0.368	0.665	0.561	0.599	0.669
52	0.264	0.345	0.302	0.502	1.032	0.607	0.613
11	0.233	0.386	0.308	0.650	0.907	0.654	0.658
18	0.633	0.402	0.526	0.465	0.545	0.712	0.707
58	0.392	0.484	0.425	0.590	0.909	0.746	0.745
24	0.423	0.606	0.528	0.540	0.602	0.778	0.772

### Identification of a reliable screening trait of salt tolerance

**Figure 6:**
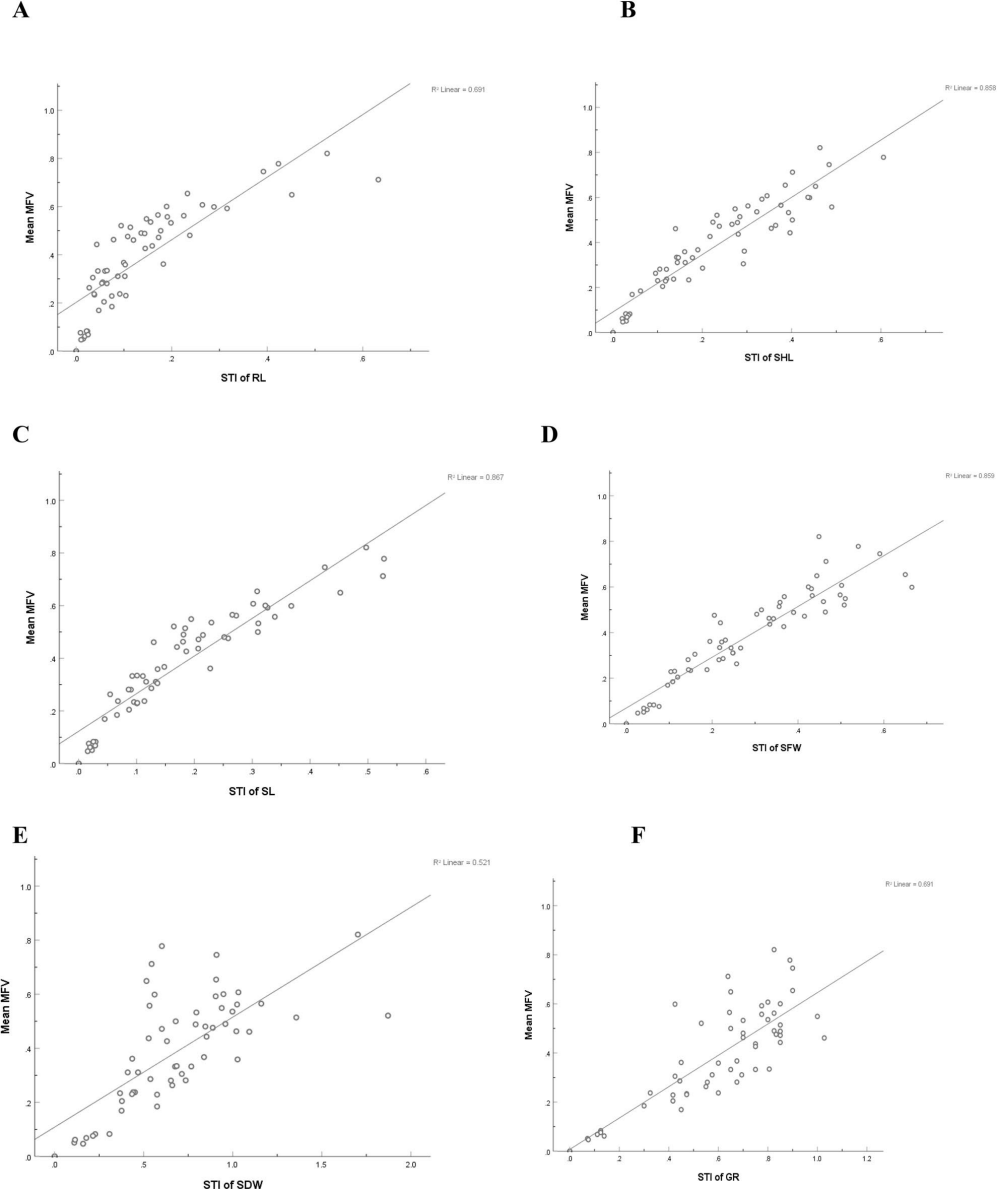
Linear correlation analysis between Salt tolerance index (STI) of each physiological parameter and Mean MFV. (**A**) Between STI of Root length and mean MFV; (**B**) between STI of Shoot length and mean MFV; (**C**) between STI of Seedling length and mean MFV; (**D**) between STI of Seedling fresh weight and mean MFV; (**E**) between STI of Seedling dry weight and mean MFV; (**F**) between STI of Germination rate and mean MFV. R^2^ Linear is the coefficient of determination.

The seedling growth parameters STIs, however, affect mean MFV indicating that mean MFV increases with an increasing STI of each seedling parameter^45^. To determine the most reliable morphological trait for estimating salt tolerance, a linear regression model is fitted with the STI and mean MFV of all morphological traits. Correlation coefficient (R^2^ = 0.859) between STI of the SFW and mean MFV (Fig. 6) was the highest (0.859) and the standardized coefficient β_SFW was also high (0.287) (Table 2) suggesting that the fresh weight of the seedling can be utilized to determine the salt tolerance of *B. juncea* genotypes at the seedling stage.

### Validation of SFW as reliable parameter

Three genotypes were selected randomly from all salt tolerance levels, and their seedling fresh weight data was recorded at optimum concentration (225 mM/L) of NaCl, to see if the parameter accurately measures salinity tolerance. There were no significant variations in growth across all genotypes in control. However, the seedling fresh weight decreased under salt stress conditions (HSS < SS < MST < ST < HST). As compared to MST and HST genotypes, which had average seedling fresh weights of 20.19 mg and 27.77 mg, respectively, HSS genotypes had an average seedling fresh weight under stress of 2.008 mg.

## Discussion

Salinity stress is one of the bottlenecks in Indian mustard yield and productivity^46^ as it hinders plant growth at the germination stage itself resulting in lower yield and biomass^47^. Developing salt tolerant cultivars has proven to be a challenging and time-consuming process as stress simulation in farm is challenging and does not provide for a robust and reproducible selection criterion which significantly impacts the efficiency of the screening procedures^48^. Expedited screening techniques should circumvent the necessity of cultivating plants under regulated circumstances and morphological characteristics serve as the foundation for effective germplasm screening protocols. Thus, different morpho physiological parameters associated with salt tolerance can serve as criteria for evaluating and selecting plants with salt tolerance at germination and early seedling stage thus saving time and effort^49,50^. Various studies have explored salinity tolerance in *B. napus* at germination stage and report shoot fresh weight and seedling fresh weight as reliable indicators for salinity stress^35,51^ but no similar research has been done in *Brassica juncea*.

In the present study, five different salt concentrations were used to screen randomly selected genotypes of *Brassica juncea* at germination and seedling stage to determine a single effective salt concentration for screening purposes. It was observed that different concentrations affect fresh weight, root and shoot length in different ways. As compared to control, higher salt concentrations tend to significantly decrease the values of traits such as root, shoot and fresh weight while lower doses tend to show a slight increase^42^. The effects of salinity have been observed to have a significant effect on growth of root and shoot in sensitive genotypes but only to a lesser extent on tolerant ones. Sensitive genotypes shown marked reduction in dry matter accumulation due to restrained leaf area development^52^. The osmotic stress may be the reason of the early growth decrease in response to salt^13^. Significant reduction in leaf area, stem and root dry weight has been reported in a comparative study among a tolerant and sensitive Indian mustard cultivars where, salinity-tolerant cultivar showed less losses in combined dry weight and dry weights of plant components such as root and shoot^40^.

Utilizing either SII or STI has proven advantageous for investigating salt stress at both the phenotypic and molecular levels^35,36,53^ therefore on the basis of STI value we found that 225 mM/L can be used as an effective concentration to screen for salt tolerance. Subsequently, tolerance of all the genotypes was determined under 225 mM/L NaCl concentration for same parameters and analyzed using the mean membership function value (MFV). The membership function value in fuzzy set theory provides an extensive analysis by employing membership functions based on fuzzy mathematics theory and the salinity-tolerant indices (STI) of observed parameters^33^ and has been successfully used for salt tolerance evaluation in rapeseed^34,35,51^, mustard^42,43^, wheat^54^, sweet sorghum^45^, sunflower^36^, rice^37^ and chickpea^38^. All genotypes were divided into 5 categories, where in,4 genotypes were classified as HST, 6 genotypes as ST, 19 genotypes as MST, 21 genotypes as SS, and 9 genotypes as HSS (Fig. 4). The maximum mean MFV was 0.820 indicating that salinity has a bigger influence on seed germination rates across the genotypes. MFV is an important indicator for the evaluation of salt tolerance^36,54^, the higher the mean MFV value, the higher the salt tolerance. Some of the genotypes exhibited lower mean MFV, indicating they have high salt sensitivity at the germination stage. Plants under salt stress experience ion stress, osmotic damage, and a buildup of reactive oxygen species. It has been anticipated that the genotypes which are salt tolerant may have efficient reactive oxygen species scavenging mechanism and are able to synthesize osmoprotectants^45^.

A mathematical formula based upon the multiple regression analysis was used for the reliable and efficient evaluation of salt tolerance in *B. juncea* lines^35^. The mathematical model was proved to be the time saving and convenient model for screening salt tolerance. The Y value was estimated for all the lines (Table S6) and used for the accurate evaluation of salt tolerance using the model. The highest value of Y depicted the higher salt tolerance which is in accordance with the results obtained for screening of salt tolerant *B. napus* lines^35^. Correlation analysis and linear fit model were used to find the relationship between various morphological parameters under NaCl stress based on the STI values of GR, RL, SHL, SL, SFW and SDW. According to the results of the correlation analysis, the STIs of SFW under the 225 mM/L NaCl treatment presented high correlation coefficients with STI of SHL and STI of SL (0.820 and 0.816, respectively). The linear fit model showed that STI of SFW had high coefficient of determination with mean MFV (R^2^ = 0.859). Thus, SFW can be used as a reliable trait for screening salt-tolerant *B. juncea* genotypes on a large scale during the germination stage. In an assessment of Indian mustard’s early responses to salinity, kinetics of germination involving root length, shoot length and fresh weight highlighted that salinity has a strong negative correlation with fresh weight^39,55^. The shoot weight along with the root length and hypocotyl length were reported to be the most significant traits to find the genetic variability governing the salt tolerance among *B. juncea* varieties which is in accordance with this study where seedling fresh weight was found to be the most significant trait for screening of salt tolerance^42^.

## Conclusion

To establish a reliable and accurate screening methodology for salt tolerance at the germination stage, a mathematical model was assessed and tested. Analysis of different growth parameters at germination stage across multiple salt stress concentrations using MFV revealed that seedling fresh weight (SFW) at an optimum concentration of 225 mM/L NaCl is the most reliable feature for screening salt tolerance in *B. juncea*. Better germination kinetics, which control biomass accumulation efficiency in developing seedlings, leads to successful earlier seedling establishment and Seedling fresh weight (SFW) might be seen as these combined processes' ultimate result. Rapid germination and strong development of seedlings increase the plant's salt resistance barrier, which is essential for sustaining plant growth and production potential. The model was used to assign a grade to each of the 59 different *B. juncea*, resulting in the following breakdown: 9 HSS, 4 ST, 19 MST, 21 SS, and 4 HST. Rapid, reproducible, and inexpensive identification of salt tolerant genotypes have direct implications in helping breeders in selection of parents in hybridization programs and developing salinity tolerant cultivars.

### Supplementary Information

Supplementary Information.

## Data Availability

The authors confirm that the data supporting the findings of this study are available within the article and its supplementary materials**.**
